# Google analytics of a pilot study to characterize the visitor website statistics and implicate for enrollment strategies in Medical University

**DOI:** 10.1186/s12909-020-02373-1

**Published:** 2020-12-01

**Authors:** Szu-Chieh Chen, Thomas Chang-Yao Tsao, Ko-Huang Lue, Yafang Tsai

**Affiliations:** 1 Department of Public Health, Chung Shan Medical University, Taichung, 40201 Taiwan, Republic of China; 2 Department of Family Medicine, Chung Shan Medical University Hospital, Taichung, 40201 Taiwan, Republic of China; 3 School of Medicine, Chung Shan Medical University, Taichung, 40201 Taiwan, Republic of China; 4 Department of Medical Quality Management, Chung Shan Medical University Hospital, Taichung, 40201 Taiwan, Republic of China; 5 Department of Pediatrics, Chung Shan Medical University Hospital, Taichung, 40201 Taiwan, Republic of China; 6 Department of Health Policy and Management, Chung Shan Medical University, Taichung, 40201 Taiwan, Republic of China; 7 Department of Medical Management, Chung Shan Medical University Hospital, No 110, Sec 1, Jianguo N, Rd, Taichung City, 40201 Taiwan, Republic of China

**Keywords:** Google analytics, Higher education, Web performance, Enrollment, Institutional research (IR)

## Abstract

**Background:**

Taiwan’s colleges and universities are struggling to maintain their student enrollment rates owing to the declining fertility rate. Focusing on students in higher education programs, this study aims to analyze online behavioral patterns for university departmental websites and accordingly, suggests response strategies to increase the rate of enrollment.

**Methods:**

We use Google Analytics to examine the websites of two departments in a medical university between February 1 and July 30, 2018. We study website patterns during the study periods for three college admission routes: STARS program, personal applications, and admission through examination and placement.

**Results:**

Most website visitors during the three visiting date ranges for the two departments are 18–24 years. The visitor groups are mainly freshmen at the university and their parents. The homepage and Subject Credits, Course Planning, Teacher Lineup, and Certificate of Subjects were the most visited webpages. The overall number of daily page views varied by academic event.

**Conclusions:**

University departments should enhance the presentation of featured courses on their webpage or distinguish course characteristics from those of competing departments in the curriculum to ensure clear market segmentation. In addition, departments should consider examining online data to identify suitable high schools that can be visited to attract potential students and to improve students’ willingness to choose their university.

**Supplementary Information:**

**Supplementary information** accompanies this paper at 10.1186/s12909-020-02373-1.

## Background

The field of institutional research (IR) was developed more than 50 years ago to support the improvement of post-secondary institutions through data-informed decision making and scholarly research in the United States [1]. Today, IR offices are rapidly growing in Taiwanese universities because of the significance assigned to them by Taiwan’s Ministry of Education (MOE). Following the growth of the Association for IR (AIR) and the increasing global presence of IR in higher education, the Taiwan Association for IR (TAIR) was established in 2016 [2].

Taiwan’s colleges and universities are grappling with the enrollment of new students because of the declining fertility rate over the past decade. In an article titled, *Falling Population Squeezes Taiwan’s Universities*, Taiwan Business TOPICS states, “Taiwan’s population is projected to peak at 23.61 million in 2021 and then begin to decrease. The fertility rate of an average of 1.17 children born to each woman is insufficient to maintain the present population level … The potential impact of a declining population on the nation’s education system is bound to be enormous, particularly at the university level where there is already a large gap between supply and demand. The Ministry of Education (MOE) estimates that the number of college students will decline by 40% to reach 723,000 by 2028” [3].

Google Analytics (GA), a free service launched by Google in November 2005, allows webmasters to access webpage content recorded by Google [4]. It enables website proprietors with limited budgets, time, and expertise to collect user data through web analytics. Organizations can, thus, obtain relevant information to analyze user behaviors or traits and derive useful marketing intelligence.

Previous studies have adopted GA and provided enterprise management with business performance insights [5–9]. Budd [5], for example, claims that website data collection can help companies develop marketing strategies to enhance business performance and further improve them to reach a wider market [6]. Applying GA to a tourism website in Vienna, Gunter and Onder [7] explore the website’s traffic data and predict travel demand. Plaza [8] claims that performance evaluations are key in enhancing the effectiveness of online marketing in the context of travel websites. The author analyzes the effectiveness of a website on the basis of traffic, access behavior, session length, browser choice, and search engines. Gordon et al. [9] use GA to assess the effectiveness of call-to-action campaigns related to kidney transplants by measuring visits, visit duration, bounce rate, number of visits, most visited webpages, user features, and usage media.

Several studies have applied GA to collect information for academic institutions. Fang [10], for example, use GA to analyze online visitor information for the Rutgers-Newark University Law Library. The findings show that librarians used different tools to create the website, and a web-based interactive learning platform improved usability for the library’s visitors by facilitating the borrowing of books and increased student usage of the school library collections. GA also provides information on visitors’ geographical location, pages visited, duration of page browsing, depth of site navigation, further navigation on the website, and end of visits [10]. Turner [11] uses GA to develop a web-based messaging application that provides behavioral, demographic, and technical information. University libraries use internet applications to improve their service efficiency and satisfaction. Braender et al. [12] develop an online learning community platform using a web-based technology at the School of Management at the College of New Jersey (TCNJ) to promote students’ social issues and cultivate student ethics. The authors further use the free GA technology to collect behavioral information about students through the learning platform and analyze the key factors influencing their learning environment.

This study examines website data collected for students enrolled in higher education programs at a Taiwanese university to obtain network marketing intelligence for university websites. It explores aspects prioritized by visitors and the redesign of website information to maximize visitor use and engagement with the studied departmental websites. It concludes with implications for early response strategies to increase student enrollment rates at universities.

## Methods

### Research target

The case school is one of the three major medical universities in central Taiwan and has five colleges and 20 departments. The gender ratio of male to female in the school is 39:61. We analyze two departments affiliated with the School of Health Management: leisure and sports (Department A) and medical hygiene (Department B). The annual enrollment quotas approved by the MOE for departments A and B are 40 and 45.

Students can choose from three admission routes recognized by MOE: STARS program (STARS), personal application (PA), and admission based on examination and placement (AE&P) [13]. The STARS program aims to provide all public and private high-school students across the country with an equal opportunity to attend college. As admission criteria, it utilizes students’ grade point average for the first 2 years of high school and the General Scholastic Ability Test (GSAT) score. All candidates must be recommended by their high schools, and a limited number of students can gain admission into college through the program.

Under the PA process, high school students apply to their preferred college or department. Students must take the GSAT and determine the eligibility of their application on the basis of the college’s criteria. Students who qualify are invited by the college to participate in the second stage of screening, during which they must take additional tests administered by the department, prepare a portfolio, and attend interviews. Students generally opt for AE&P when they have failed to gain admission through the STARS or PA processes or are dissatisfied with their results. Under the AE&P process, students must take the advanced subjects test (AST), after which they fill out a preference list indicating their preferred colleges and departments.

### Enrollment stages

This study divided the PA route into two periods, pre- and post-PA, to conduct a comparative analysis. The pre-PA period was that before the registration deadline on February 26, 2018. During this stage, candidates sought information about the department online. The post-PA period includes announcement of results by the selection committee on March 23, 2018, and that of test dates between April 11 and 29, 2018 for designated projects in each school. During this period, students visited the university website to familiarize themselves with the department as well as its teachers and future direction. The definition of this period is based on a student’s application schedule for admission to the university (see Table 1 in the Additional file 1: Appendix).

**Table 1:** Time-series of page views during enrollment period for the AE&P route in the two departments. Three periods for admission by AE&P were (1) July 19 to July 23, 2018, (2) July 24 to July 28, 2018 and (3) July 29 to August 7, 2018)

Department A				Department B			
Date	Program	Courses and Credits	Faculty	Date	About us	Programs	Courses and Credits
2018/7/19	44	10	28	2018/7/19	55	41	25
2018/7/20	10	15	23	2018/7/20	32	23	7
2018/7/21	12	7	1	2018/7/21	53	18	14
2018/7/22	13	18	5	2018/7/22	24	22	10
2018/7/23	19	29	10	2018/7/23	56	41	24
SUM	98	79	67	SUM	220	145	80
%	40%	32%	28%	%	49%	33%	18%
2018/7/24	18	12	24	2018/7/24	48	51	29
2018/7/25	26	18	24	2018/7/25	36	50	30
2018/7/26	24	13	13	2018/7/26	30	46	28
2018/7/27	48	29	15	2018/7/27	47	35	21
2018/7/28	19	7	4	2018/7/28	25	22	10
SUM	135	79	80	SUM	186	204	118
%	46%	27%	27%	%	37%	40%	23%
2018/7/29	0	0	2	2018/7/29	7	6	6
2018/7/30	21	3	13	2018/7/30	6	3	2
2018/7/31	5	8	15	2018/7/31	6	4	1
2018/8/1	2	4	21	2018/8/1	6	1	0
2018/8/2	5	19	17	2018/8/2	7	1	0
2018/8/3	5	15	5	2018/8/3	6	1	0
2018/8/4	4	7	17	2018/8/4	9	4	0
2018/8/5	4	5	16	2018/8/5	10	6	0
2018/8/6	9	4	3	2018/8/6	6	2	3
2018/8/7	80	27	34	2018/8/7	62	33	12
SUM	135	92	143	SUM	125	61	24
%	36%	25%	39%	%	60%	29%	11%

For AE&P, the designated enrollment period is divided into three parts (see Table 2 in the Additional file 1: Appendix). The first period was the announcement of the AST grades between July 19 and 23, 2018. During this period, the students filled out enrollment quotas and registered for stand-alone electives, and the departments announced designated subject examinations. In the second period, from July 24 to 28, 2018, students filled out a preference list on the website (PLW) and registered on the network, and the colleges assigned electives to the students. The third period, that is, July 29–August 7, 2018, entailed the announcement of placement results (APR) and an admission notice for university entrance examinations.

**Table 2:** The depth of visit is a measure of visit quality

Department A			Department B		
Page subject	Page view (%)		Page subject	Page view (%)	
	Before PA	After PA		Before PA	After PA
Visit depth (initial pages)					
Main website	**679 (81%)**	**1221 (87%)**	Main website	**1185 (68%)**	**1155 (63%)**
About us	79 (10%)	77 (6%)	About us	350 (20%)	314 (17%)
Courses and credits	31 (4%)	21 (2%)	News and events	116 (7%)	204 (11%)
Program	21 (3%)	18 (1%)	Faculty	99 (6%)	150 (8%)
International conference and exhibition program area	18 (2%)	36 (3%)			
HACCP	12 (1%)	35 (3%)			
**Total views**	**840**	**1408**	**Total views**	**1750**	**1823**
Visit depth (first pages)					
Courses and credits	**132 (47%)**	80 (26%)	About us	**302 (42%)**	80 (21%)
Program	64 (23%)	58 (19%)	Program	199 (28%)	58 (15%)
About us	45 (16%)	**89 (29%)**	Faculty	101 (14%)	**89 (24%)**
Main website	24 (9%)	28 (9%)	Aim	53 (7%)	28 (7%)
Brief history	18 (6%)	53 (17%)	News and events	34 (5%)	61 (16%)
			Admission	25 (4%)	61 (16%)
**Total views**	**283**	**308**	**Total views**	**714**	**377**
Visit depth (second pages)					
Main website	**119 (51%)**	**192 (63%)**	Main website	**124 (29%)**	**134 (37%)**
Courses and credits	56 (24%)	25 (8%)	Courses and credits (2017)	81 (19%)	40 (11%)
About us	32 (14%)	35 (12%)	Courses and credits (past)	73 (17%)	43 (12%)
Program	17 (7%)	23 (8%)	Program	62 (15%)	39 (11%)
Brief history	9 (4%)	29 (10%)	About us	52 (12%)	65 (18%)
			Faculty	31 (7%)	44 (12%)
**Total views**	**233**	**304**	**Total views**	**423**	**365**
Visit depth (third pages)					
Program	**27 (27%)**	30 (18%)	Courses and credits (2017)	**86 (35%)**	**54 (27%)**
About us	24 (24%)	**60 (36%)**	Program	66 (27%)	48 (24%)
Courses and credits	22 (22%)	20 (12%)	About us	36 (15%)	41 (20%)
Main website	21 (21%)	30 (18%)	Courses and credits (past)	34 (14%)	20 (10%)
Features of the department	5 (5%)	25 (15%)	News and events	26 (10%)	39 (19%)
**Total views**	**99**	**165**	**Total views**	**248**	**202**

We listed the page views of the different page subjects for the initial, first, second, and third pages visited. Before PA was from February 26 to March 22, 2018; After PA was from March 23 to May 17, 2018

### Google analytics

This study uses GA to obtain site usage data from February 1 to July 30, 2018. Google collects and links actions through a JavaScript code installed on the webpage. An analysis of the GA data on visitor behaviors highlighted information that visitors expect to receive through the faculty page, which can serve as a reference for departments to better design their webpages and plan the curriculums. In addition, identifying content that interests online visitors allows departments to more effectively explore and meet the requirements of potential students. GA provides data for the following metrics: number of page views, duration of visit, pages most often visited, depth of visit, user demographics (e.g., gender, age, city, and country), and user media (e.g., type of device used).

## Results

### Department characteristics

**Fig. 1:**
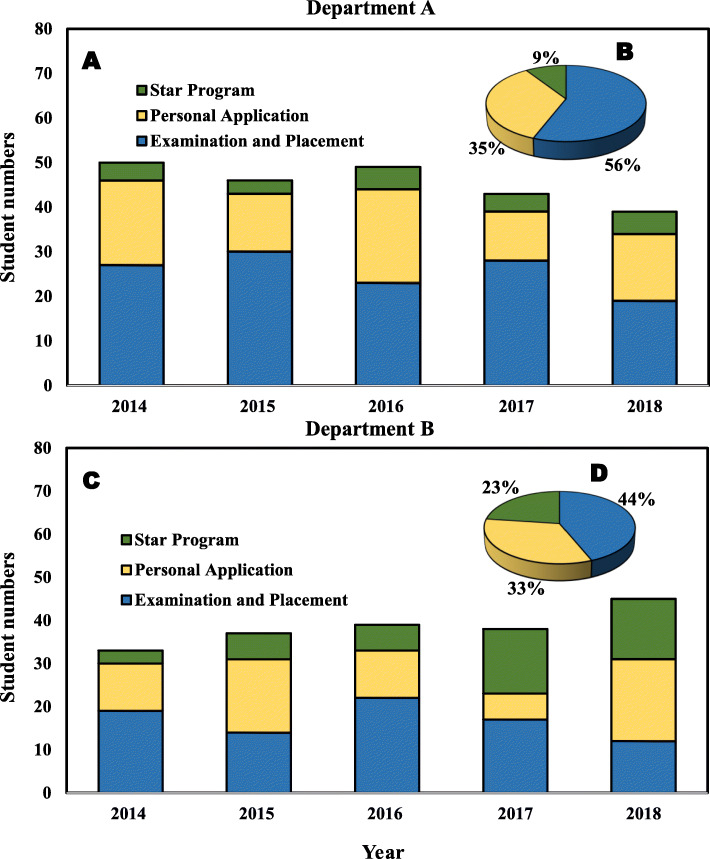
Student enrollment rate and admissions routes for 2014–2018 in (**a**) Department A and (**b**) Department B. Percentage of students who enrolled through the STARS Program (SP), personal application (PA), and admission by examination and placement (AE&P) in (**c**) Department A and (**d**) Department B

Figure 1a and c depict the student enrollment rate and admission routes for departments A and B during 2014–2018. The numbers of students enrolled in departments A and B increased from 33 to 45 and from 39 to 53 over a five-year period. The numbers differ from the annual approved quota because we do not consider independent enrollment, international exchange students, or transfer students and analyze data only for local or national students. Figure 1b and d show the overall percentage of students enrolled through STARS, PA, and AE&P in the two departments. The three admissions channels, respectively, account for 9, 35, and 56% of the total enrolled rate for Department A and 23, 33, and 44% of the rate for Department B. Evidently, AE&P is the main enrollment route in both the departments.

**Fig. 2:**
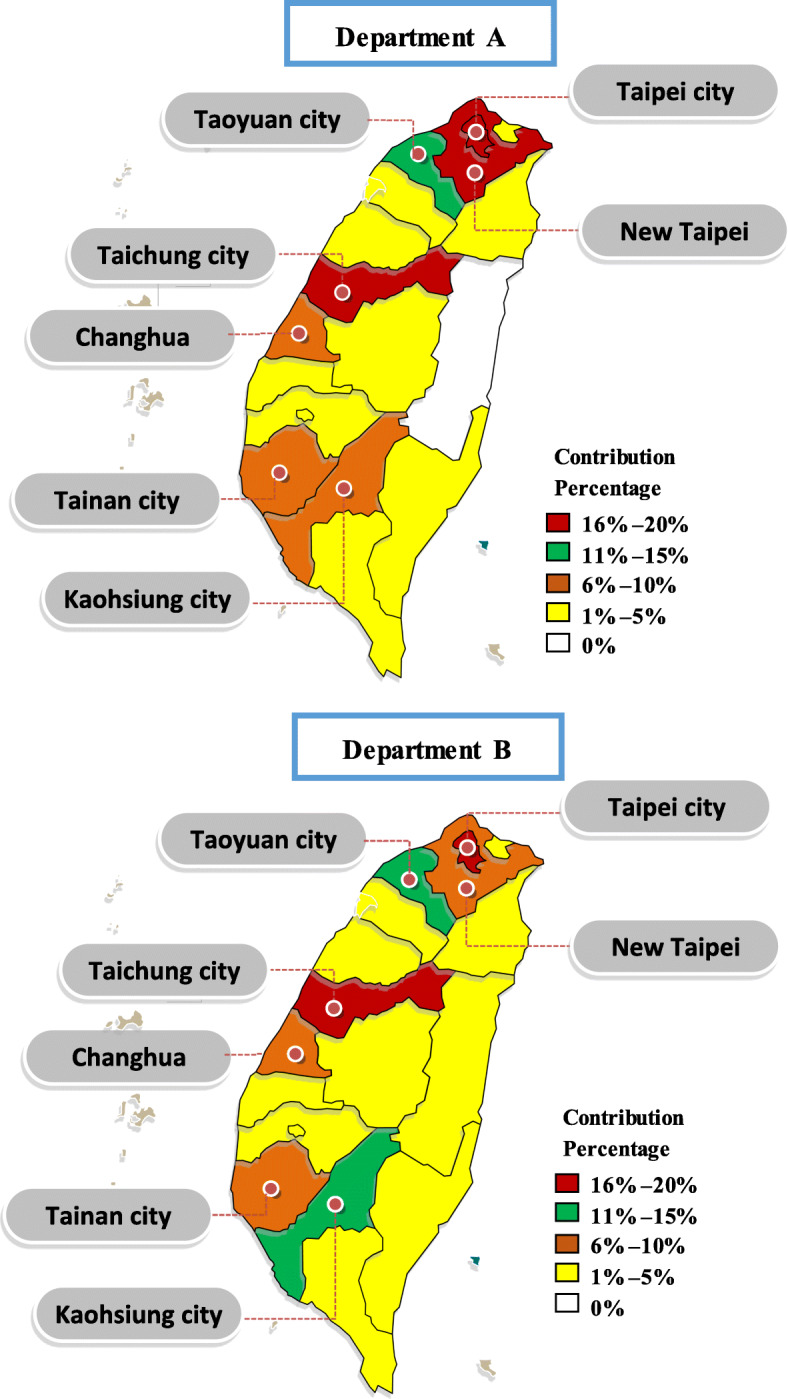
Geographic distribution of student visitors

Figure 2 presents the geographic distribution of students. A majority of the students enrolled in Department A are from a metropolitan area, with 16–20% from Taipei, New Taipei, and Taichung cities and 11–15% from Taoyuan City. For Department B, 16–20% of the students are from Taipei and Taichung cities and 11–15% are from Taoyuan and Kaohsiung cities.

### Website profile during PA stage

This study analyzes the time-series data for page views between the pre- (February 26–March 22, 2018) and post-PA (March 23–May 17, 2018) periods for the two departments (see Table 3 in the Additional file 1: Appendix). The average daily browsing durations in the pre-PA period were marginally higher than those in the post-PA period. The average page views per day during the entire study period were 39 and 27 for departments A and B. Department B’s page views per day in the pre- and post-PA periods are 87 and 35.

**Table 3:** We listed the page views of the different page subjects for initial, first, second, and third pages. The three phases for the duration of the AE&P were July 19 to 23, 2018 (announcement of grades for AST), July 24 to 28, 2018 (fill out a PLW), and July 29 to August 7, 2018 (APR)

Department A				Department B			
Page subject	Page view (%)			Page subject	Page view (%)		
	AST	PLW	APR		AST	PLW	APR
Visit depth (initial pages)							
Main website	**27 (44%)**	0 (0%)	**76 (67%)**	Main website	**234 (57%)**	**293 (64%)**	**208 (58%)**
About us	17 (27%)	16 (30%)	7 (6%)	About us	92 (22%)	73 (16%)	65 (18%)
Program	11 (18%)	**21 (39%)**	10 (9%)	Program	46 (11%)	58 (13%)	11 (3%)
Admission	3 (5%)	0 (0%)	2 (2%)	Courses and credits	13 (3%)	7 (2%)	5 (1%)
Courses and credits	2 (3%)	9 (17%)	4 (4%)	Admission	13 (3%)	16 (4%)	40 (11%)
Faculty	2 (3%)	5 (9%)	7 (6%)	Faculty	12 (3%)	10 (2%)	29 (8%)
Brief history	0 (0%)	3 (6%)	7 (6%)				
**Total views**	**62**	**54**	**113**	**Total views**	**410**	**457**	**358**
Visit depth (first pages)							
Courses and credits	**19 (29%)**	**32 (34%)**	20 (20%)	About us	**63 (40%)**	58 (32%)	17 (16%)
Program	16 (24%)	30 (32%)	**26 (26%)**	Program	44 (28%)	**68 (37%)**	18 (17%)
Brief history	16 (24%)	15 (16%)	15 (15%)	Courses and credits	13 (8%)	14 (8%)	2 (2%)
Faculty	10 (15%)	10 (11%)	19 (19%)	Main website	12 (8%)	8 (4%)	6 (6%)
Admission	5 (8%)	6 (7%)	19 (19%)	Faculty	11 (7%)	20 (11%)	**52 (48%)**
				Career information	6 (4%)	16 (9%)	0 (0%)
				New student	9 (6%)	0 (0%)	14 (13%)
**Total views**	**66**	**93**	**99**	**Total views**	**158**	**184**	**109**
Visit depth (second pages)							
Program	**25 (38%)**	9 (15%)	11 (13%)	Main website	**24 (25%)**	**38 (27%)**	**31 (53%)**
Courses and credits	12 (18%)	16 (27%)	11 (13%)	Program	20 (21%)	27 (19%)	7 (12%)
Brief history	14 (21%)	5 (8%)	9 (10%)	Courses and credits (past)	17 (18%)	29 (21%)	7 (12%)
Faculty	10 (15%)	2 (3%)	11 (13%)	Courses and credits (2017)	16 (17%)	25 (18%)	2 (3%)
Main website	0 (0%)	**23 (38%)**	**41 (47%)**	About us	13 (14%)	15 (11%)	6 (10%)
Features of the department	5 (8%)	5 (8%)	5 (6%)	Faculty	5 (5%)	5 (4%)	6 (10%)
**Total views**	**66**	**60**	**88**	**Total views**	**95**	**139**	**59**
Visit depth (third pages)							
Program	**11 (33%)**	**10 (37%)**	11 (20%)	Courses and credits (2017)	**18 (32%)**	**38 (40%)**	7 (20%)
Courses and credits	7 (21%)	3 (11%)	4 (7%)	Program	14 (25%)	21 (22%)	5 (14%)
Main website	4 (12%)	6 (22%)	**12 (22%)**	Courses and credits (past)	9 (16%)	19 (20%)	6 (17%)
License information	4 (12%)	7 (26%)	6 (11%)	About us	9 (16%)	3 (3%)	3 (9%)
Faculty	4 (12%)	8 (30%)	**12 (22%)**	Faculty	3 (5%)	9 (10%)	5 (14%)
Brief history	3 (9%)	2 (7%)	10 (18%)	Admission	3 (5%)	4 (4%)	**9 (26%)**
**Total views**	**33**	**36**	**55**	**Total views**	**56**	**94**	**35**

The data also include the percentage of students visiting each link on the page (see Table 3 in the Additional file 1: Appendix). For Department A, withdrawn webpages received the highest number of visitors in the pre- (36%) and post-PA (49%) periods, followed by About Us and Courses and Credits. Withdrawn webpages are pages that were unavailable during the data analysis because the departments removed them from the website. The unavailable pages include past news, announcements, guest lecturers, and notifications of limited website capacity. In the case of Department B, the About Us page received the highest number of visitors during both the pre- (44%) and post-PA (42%) periods, followed by the pages of Faculty (26%) and Programs (25%). The number of daily page views varied by admissions event; for example, the highest number of page views was observed on April 14, 15, 21, and 22, 2018, which corresponded with the dates of interviews held at the university.

### Website profile during AE&P stage

Table 1 presents the time-series data on page views during the enrollment periods for the AE&P route. For Department A, the number of visitors on the Course Information and Certificate of Subjects pages peaked following the announcement on July 19, 2018. Before registering for an elective, students generally seek an overview of the course information and subject credits. The online registration for and the distribution of electives was from July 24 to 28, 2018. During this period, visitors also navigated the Teachers page. The results reveal that the Faculty page gains priority during electives registration, particularly at the end of the registration day. The Faculty page received the highest traffic, followed by the Program page. Thus, the departments should update their course information before announcing the assessment results, and their curriculum planning can be associated with students’ choice of electives (Table 1). For Department B, the average daily visits during the three stages of AE&P were 89, 102, and 21, respectively. The highest number of overall page views was recorded during the second stage, with the About Us page receiving the highest number of visitors, followed by the Program page (Table 1).

### Webpage interaction information

Table 2 shows the depth of visits on the university’s website. For Department A, the homepage received the highest initial clicks, accounting for 81 and 87% of the total number of clicks in the pre- and post-PA period. From the homepage, 47% visitors clicked on Courses and Credits and 29% clicked on About Us. Thereafter, most visitors returned to the homepage, accounting for 51 and 63% of the total number of clicks in the pre- and post-PA period, before proceeding to Program (27%) and About Us (36%) in the pre- and post-PA periods. We speculate that once the students confirm their admission, they seek a more comprehensive understanding of the department. The number of visitors who continued to browse the website after visiting the three pages (Courses and Credits, About Us, and Program) dropped to 12% during both the periods. For Department B as well, the homepage received the highest number of initial clicks, accounting for 68 and 63% of the total number of visitor during the pre- and post-PA periods. The total views in the pre-PA period decreased from 1750 to 248, with only 14% visitors remaining, and those in the post-PA period decreased from 1823 to 202, with 11% visitors remaining. This suggests that visitors tend to leave the website once they have obtained the necessary information (Table 2).

Table 3 presents the page views for the webpages of each subject. A majority of the visitors for Department A initially clicked on the homepage before proceeding to Courses and Credits, returning to the homepage, and then visiting the Program page. The results for Department B slightly differ among the AST, PLW, and APR stages. Following the results announcement, a majority of the visitors clicked on the homepage (57%) and then the About Us page (40%), following which they returned to the homepage (25%) and finally clicked on the Courses and Credits page (32%). For the network registration and distribution of electives (PLW) stage, visitors began on the homepage and then clicked on the Program and Faculty pages. After interacting with the three pages, the total views decreased from 410 to 56, leaving only 14% visitors, while the remaining percentages were 21 and 10%.

For Department A, the homepage and the Subject Credits and Course Planning pages received the highest number of visits. For Department B, the visitors began on the homepage and then proceeded to the Introduction and Teacher Lineup pages before returning to the homepage and then further visiting the Certificate of Subjects page. After interacting with the three webpages, the page views for departments A and B were 12 and 13% (Table 2) and 56 and 15% (Table 3), indicating that the visitors exited the website upon obtaining the necessary information.

### User characteristics

Table 4 presents user information and characteristics. For Department A, most visitors were 18–24 years during the three time periods, followed by those aged 25–34 years. The visitor groups were mainly freshmen at the university, followed by their parents. In terms of browsing tools, most visitors used desktops, followed by smartphones. Thus, the webpage design should meet the usage requirements of both desktop computers and smartphones. For Department B, the number of female visitors was marginally higher than male visitors. The age group accounting for the highest number of visitors was 18–24 years, followed by 45–54 years. GA also offers visitors’ geographic and demographic data. These findings suggest that the website design should be women centric given the higher number of female visitors.

**Table 4:** User Information and Characteristics for Department A and Department B

Variables	Department A				
	Before PA	After PA	AST	PLW	APR
Gender	ND	ND	Female (65%)	Female (60%)	Female (75%)
Age	ND	ND	18–24 years (28%) 45–54 years (22%)	18–24 years (34%) 45–54 years (31%)	18–24 years (50%) 25–34 years (47%)
Location	Taoyuan city (44%) Changhua county (30%)	Taoyuan city (28%) Taipei city (13%) Changhua county (12%)	Taoyuan city (37%) Changhua county (14%) Hsinchu county (20%)	Taoyuan city (36%) Changhua county (16%) Tainan city (10%)	Taoyuan city (37%) Changhua county (11%)
Devices	Desktop (55%) Mobile (43%)	Desktop (55%) Mobile (43%)	Desktop (61%) Mobile (38%)	Desktop (57%) Mobile (40%)	Desktop (63%) Mobile (44%)
Department B					
Gender	ND	ND	Female (58%)	Female (57%)	Female (54%)
Age	ND	ND	18–24 years (46%) 45–54 years (33%)	18–24 years (55%) 45–54 years (28%)	18–24 years (49%) 25–34 years (27%)
Location	Taoyuan city (39%) Hsinchu county (11%)	Taoyuan city (31%) Hsinchu county (14%) Changhua county (13%)	Taoyuan city (52%) Changhua county (6%)	Taoyuan city (42%) Changhua county (9%)	Taoyuan city (55%) Changhua county (11%)
Devices	Mobile (50%) Desktop (48%)	Desktop (53%) Mobile (44%)	Desktop (50%) Mobile (47%)	Desktop (56%) Mobile (41%)	Desktop (53%) Mobile (44%)

### Average page time

**Fig. 3:**
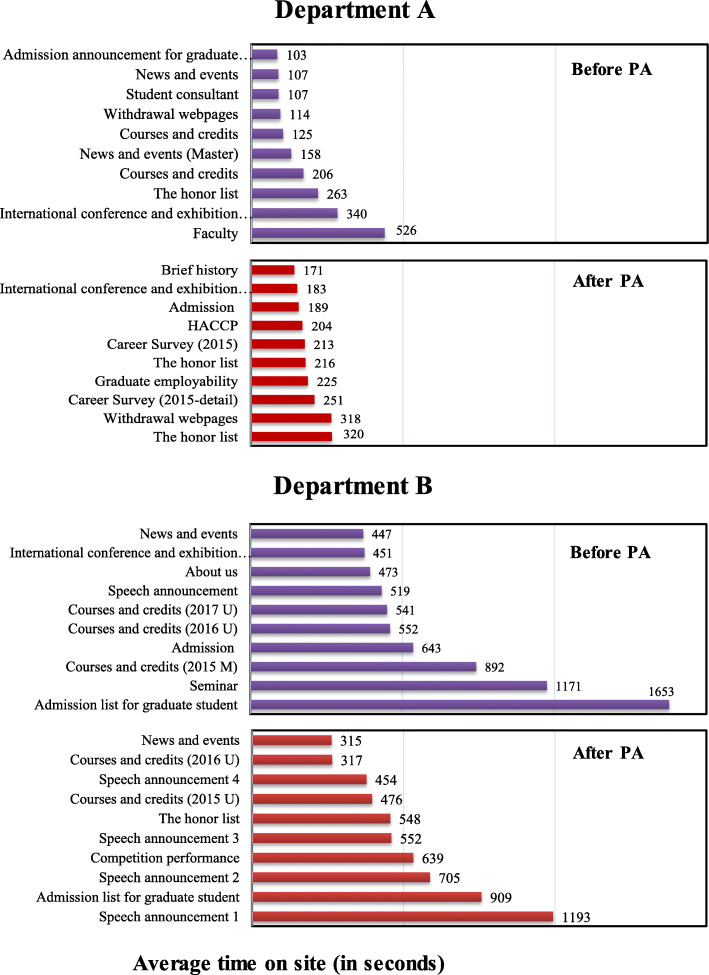
Average time spent on site (in seconds) pre- and post-PA

Figure 3 depicts the average time spent on the site (in seconds) in the pre- and post-PA periods. For Department A, visitors spent most of their time on the Faculty page, while for Department B, they spent a majority of their time browsing the admissions list for graduate students. These findings suggest that some visitors are students of the institute.

**Fig. 4:**
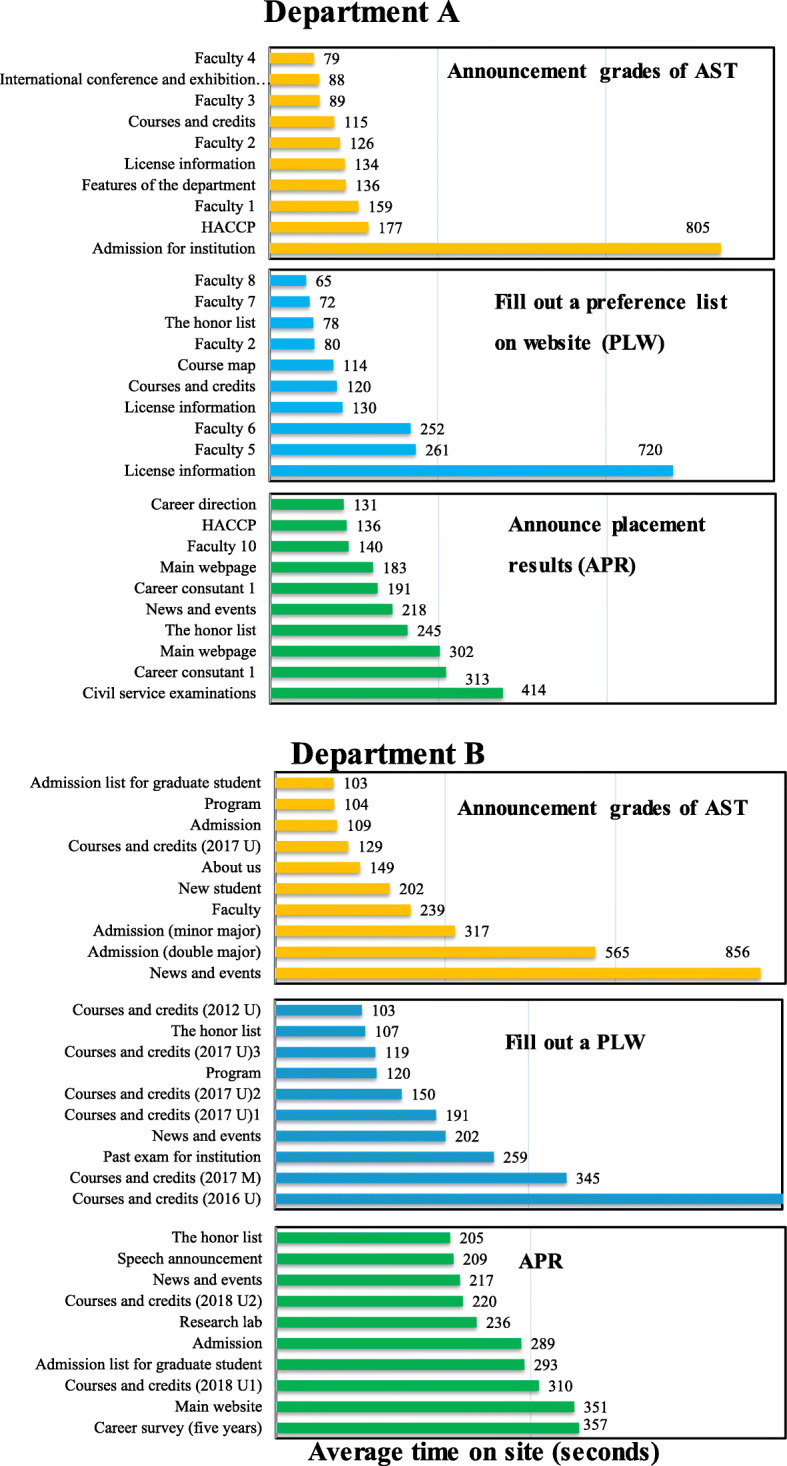
Average time spent on site (in seconds) during three stages: announcement of grades for advanced subjects test (AST), filling out of a preference list on website (PLW), and announcement of placement results (APR)

Figure 4 shows the average time spent on the site (in seconds) during the AST, PLW, and APR stages. For Department A, the total browsing time is the longest for the Admissions Announcement for the master’s class, followed by the HACCP Course Admissions Guide, in the 5 days before the AST results were announced. The overall browsing time during the PLW phase indicates that visitors spent most of their time browsing license information. In the APR phase, the longest browsing time is reported for Study Program, followed by Food Technician Exam. Overall, the website visitors sought information on the selection of electives on the Review Information and Training Qualifications pages. Thus, it is important that the university maintains an updated list of examinations. Visitors also browsed teachers’ professional background and items of concern. For Department B, visitors spent more than 5 minutes browsing the latest news, enrollment status (double major and adjunct), subject and credits table, and graduate flow tracking (five-year) survey.

The average time visitors spent on the webpage during Department B’s recommendation stage is about twice that of the assessment period. However, the findings are directly inversed for Department A (see Figs. 3 and 4), that is, the two pages on which visitors stayed the longest differ. Department A is a resting and kinematics group that focuses on five-year follow-up surveys and subject credits. Department B, on the other hand, is a medical and health group. Visitors stayed the longest on the Investigation List, Graduation Topics, and Master’s Degrees Credits Tables pages, indicating that students seek information during the stages of university referrals and entrance exams. The visitors are potential candidates for the master’s class. During the assessment phase, visitors stayed the longest on the Certificate of Subjects (University) and Graduate Flow Tracking pages.

### Visitor location

For Department A, the locations of visitors were similar during the recommendation and assessment stages (i.e., Zhongli, Taoyuan, and Changhua). For Department B, a majority of the visitors were from Taoyuan City and Changhua County.

## Discussion

This study uses GA to examine network behavioral patterns demonstrated by students visiting university department websites. The gathered marketing intelligence is further analyzed to enhance website use experience and increase the student enrollment rate.

The findings for the average daily page views suggest that students strengthen their understanding of the department before their interviews, particularly when the interviews are included in the scoring system. Students’ choice of electives can influence credits and course arrangements. This research suggests that departments should enhance the presentation of featured courses on their webpages or distinguish their course characteristics from those of competing departments in the curriculum to ensure clear market segmentation. The highest network traffic was recorded when students checked their scores at the end of the registration period and received their assessment results before the distribution of electives. Thus, departments could identify aspects to promote on their website during this period, especially on the Introduction to the Department and Course Planning pages.

GA instantly captures students’ location information for each department after the application results are distributed. This is contrary to conventional means wherein students must report their location to the school once they begin the application process. The School Affairs Research Office requested the Information Room to assist the departments in installing the GA code. We recommend that once the results are distributed, each department evaluate the online information and identify suitable high schools to visit to identify potential students and to improve student recruitment. However, it is important to compare the degree of matching between GA user information and actual admissions for each department to confirm our results. This study (see Visitor Location section and Fig. 2) further shows that Taoyuan City was a common visitor location between both departments. In other words, website visitors may include students’ parents or siblings and some students who were not admitted.

The study is subject to the following limitations. First, the data metrics are limited to those provided by GA. The variables include page views, webpage interactive information, average time spent on site, page subject, and user characteristic (i.e., gender, age, location, and type of device used). We have presented results for all the above-mentioned variables, and no continuative analysis of other variables is possible. Second, the same visitor may click on a link multiple times, causing the reading rate to increase. During the data collection period, inquiries were made about the scheduling of the master’s class. However, only some readers may be potential candidates. Third, considering the school administrative unit, the departments’ processes and operations, and the influence of enrollment or activities related to other master’s programs, an analysis of representative data to determine other influencing factors is necessary. Finally, a significant number of visits were made to withdrawn pages and thus, there is uncertainty about the content.

## Conclusions

This study shows that the following pages received the highest number of visitors: homepage, Subject Credits, Course Planning, Teacher Lineup, and Certificate of Subjects. Thus, university departments should consider enhancing the presentation of featured courses on their webpages or distinguish their course characteristics from those of competing departments in the curriculum to ensure clear market segmentation. Further, each department should examine online data and identify suitable high schools to visit to attract potential students and improve student willingness to choose their university.

## Supplementary Information

**Additional file 1: Appendix 1.** Individual application project and date description (University Selection Admission Committee). **Appendix 2.** Timeline for assessment and distribution (University Entrance Examination Center). **Appendix 3.** Time-series data for page views for both departments in pre- (February 26–March 22, 2018) and post-PA (March 23–May 17, 2018) periods.

## Data Availability

The datasets used and/or analyzed in this study can be made available by the corresponding author upon reasonable request.

## Abbreviations

GA: Google Analytics
IR: Institutional research
PA: Personal application
AE&P: Admission by examination and placement
GSAT: General scholastic ability test
AST: Advanced subjects test
PLW: Preference list on website
APR: Announce placement results
