# Supplementary material for: Google analytics of a pilot study to characterize the visitor website statistics and implicate for enrollment strategies in Medical University
Source: BMC Med Educ. 2020 Dec 1;20:483. doi: 10.1186/s12909-020-02373-1 (PMC7708183; doi:10.1186/s12909-020-02373-1)
Supplement: Supplementary file 1 — Additional file 1: Appendix 1. Individual application project and date description (University Selection Admission Committee). Appendix 2. Timeline for assessment and distribution (University Entrance Examination Center). Appendix 3. Time-series data for page views for both departments in pre- (February 26–March 22, 2018) and post-PA (March 23–May 17, 2018) periods. [file 12909_2020_2373_MOESM1_ESM.docx]

**Appendix Table 1. Individual Application Project and Date Description (University Selection Admission Committee)**

| Item | | Date |
| --- | --- | --- |
| Admissions Guide Announcement | | 2017.11.01（Wed） |
| Admissions Guide for Sale | | 2017.11.09（Thu） |
| Moderate or severe hearing-impaired students are exempt from the high school English listening test qualification application and sending documents | | 2018.01.16（Tue）to 2018.01.17（wed） |
|  |  | Daily from 8 am to 5 pm |
| Selection Committee Announcement Exemption from High School English Listening Test Results | | 2018.01.29（Mon）2 pm |
| Test by University Entrance Examination Center | | 2018.01.26（Fri）to 2018.01.27（Sat） |
| Test by University Science Examination Board Federation | | Music：2018.02.08（Thu）至2018.02.11（Sun） |
|  |  | Art：2018.02.02（Fri）至2018.02.03（Sat） |
|  |  | Sports：2018.02.05（Mon）至2018.02.07（Wed） |
| University Entrance Examination Center sends a report of the results of the subject tests | | 2018.02.23（Fri） |
| University Science and Technology Examination Board Federation sends a notice of the results of the examination | | 2018.02.26（Mon） |
| High school (job) unified uploading of the enrolled subject ability test for the high school (job) school performance certificate (PDF file) | | 2018.03.15（Thu）to 2018.03.16（Fri） |
| (Non-graduate candidates should upload it themselves during the review data upload period) | | Daily from 8 am to 7 pm |
| New graduates of the enrollment subject competency test are enrolled in the high school (job) school performance certificate (PDF file) | | 2018.03.20（Tue）to 2018.03.26（Mon） |
|  |  | Daily from 8 am to 9 pm |
| 1st stage | Collective registration schools pay fees to the selection committee | 2018.03.19（Mon）From 0:00 am to |
|  |  | 2018.03.22（Thu）5 pm |
|  | Individual applicants apply for payment to the selection committee | 2018.03.19（Mon）From 0:00 am to |
|  |  | 2018.03.22（Thu）5 pm |
|  | The group registration school applies to the selection committee | 2018.03.20（Tue）to 2018.03.22（Thu） |
|  |  | Daily from 8 am to 5 pm |
|  | Individual applicants apply to the Selection Board | 2018.03.20（Tue）to 2018.03.22（Thu） |
|  |  | Daily from 8 am to 5 pm |
|  | Selection Committee Announcement Screening Results | 2018.03.28（Wed） |
|  | Screening results review deadline | 2018.03.29（Thu） before 5 pm |
| 2^nd^ stage | University send (or announce) designated project test notice and related materials | Universities make their own decisions (for details consult Schools and Departments) |
|  | Pay the designated project test fee | Universities make their own decisions (for details consult Schools and Departments) |
|  | Review data network upload or due certificate receipt deadline | Universities make their own decisions (for details consult Schools and Departments) |
|  | Designated project test date | Universities make their own decisions（2018.04.11 to 2018.04.29） |
|  | University Announcement Admissions List and Sending Selection of Total Transcripts | Universities make their own decisions（before 2018.05.07） |
|  | Review of the total score review deadline | Universities make their own decisions (for details consult Schools and Departments) |
| Unified distribution | Selection Committee sends online registration to read elective order code | 2018.04.24（Tue） |
|  | University uploads individual application admission enrollment results (including results of review results) to the selection committee | Before 2018.05.07（mon） |
|  | Enrolled students register with the selection committee to enroll in the elective order | 2018.05.10（Thu）to 2018.05.11（Fri） |
|  |  | Daily from 8 am to 9 pm |
|  | Selection Committee Announcement to Distribute Results and Report to Universities | 2018.05.17（Thu） |
|  | Unified distribution result review deadline | Before 12pm 2018.05.18（Fri） |
|  | Admissions students give up the qualifications for admission (postmarks) | 2018.05.21（mon） |
| The university report to the selection committee about the declined list | | 2018.05.29（Tue）to 2018.05.31（Thu） |
| Selection Committee to the University Examination Admissions Committee for the four technical and two professional schools | | 2018.06.06（Wed） |
| The Joint Registration and Distribution Admission Committee enrolls the student enrollment list | |  |

**Appendix Table 2. Timeline for Assessment and Distribution**

**(University Entrance Examination Center)**

| Month | Item |  |
| --- | --- | --- |
| August | 2017/08/04~2017/11/09‧ Release “Technical Examination Guide” |  |
|  | 2017/08/04~2018/07/03‧ Release “2018 Academic Examination Certificate” |  |
| September | 2017/09/04~2017/09/08‧ Registration for “High School English Listening Test (1st Exam)” |  |
| October | 2017/10/21‧ Exam – “High School English Listening Test (1st Exam)” |  |
|  | 2017/10/27~2017/11/09‧ Registration for “Technical Examination” |  |
|  | 2017/10/27~2017/11/09‧ Registration for “Principal Competency Test” |  |
| November | 2017/11/03~2017/11/09‧ Registration for “High School English Listening Test (2nd Exam)” |  |
|  | 2017/11/09~2018/07/28‧ Release “University Exam Admissions Distribution Admissions Guide” |  |
| December | 2017/12/16‧ Exam – “High School English Listening Test (2nd Exam)” |  |
| January | 2018/01/26~2018/01/27‧ Exam – “Principal Competency Test” |  |
| February | 2018/02/02~2018/02/11‧ Exam – “Technical Examination” |  |
| May | 2018/05/08~2018/05/24‧ Registration for “Specified Subject Test” |  |
|  | 2018/05/07~2018/07/28 Release “University Examination Admission Registration Information” (including important announcements, school codes, approved places and registration bills, etc.) |  |
|  |  |  |
|  | 2018/05/07~2018/07/18‧ Provide registration elective exercise version and teaching video download |  |
|  | 2018/05/08~2018/06/01‧ Certification document review |  |
|  | Announce admission results (release) |  |
| June | 2018/06/11 (9:00 am) | -- |
|  | Announce “Review results of relevant certification documents” |  |
|  | 2018/06/11~2018/06/20 (by 5:00 pm) |  |
|  | Application for acceptance of the examination document |  |
|  | 2017/06/26 (9:00 am) |  |
|  | Publish the certification document review application result |  |
| July | 2018/07/01~2018/07/03‧ Exam – “Specified Subject Test” |  |
|  | 2018/07/19 |  |
|  | Announcement of enrollment quotas (including reflow quotas), registration of elective stand-alone editions, cumulative number of students in designated subject examinations, and minimum registration standards |  |
|  | 2018/07/19 (9:00 am) ~ 2018/07/28 (till 3:00 pm) |  |
|  | Open “Payment of Registration Fee” |  |
|  | 2018/07/24 (9:00 am) ~2018/07/28 (till 4:30 pm) |  |
|  | Open “Network Registration Distribution of Electives” |  |
| August | 2018/08/07‧ University exam admission distribution announcement (released) |  |
|  | 2018/08/07~2018/08/13‧ Acceptance of distribution results review |  |
|  | 2018/08/20‧ Announce distribution review results |  |

**Appendix Table 3. Time-series of page views before (February 26 to March 22, 2018) and after PA (March 23 to May 17, 2018) in the two departments.**

| **Department A (Before PA)** | | | | **Department A (After PA)** | | | |
| --- | --- | --- | --- | --- | --- | --- | --- |
| Date | Withdrawal web pages | Courses and Credits | About us | Date | Withdrawal web pages | About us | Programs |
| 2018/2/26 | 14 | 22 | 33 | 2018/3/23 | 10 | 1 | 1 |
| 2018/2/27 | 22 | 17 | 21 | 2018/3/24 | 9 | 4 | 3 |
| 2018/2/28 | 14 | 43 | 24 | 2018/3/25 | 4 | 0 | 0 |
| 2018/3/1 | 31 | 18 | 26 | 2018/3/26 | 15 | 0 | 2 |
| 2018/3/2 | 11 | 18 | 13 | 2018/3/27 | 9 | 2 | 0 |
| 2018/3/3 | 5 | 19 | 11 | 2018/3/28 | 20 | 34 | 5 |
| 2018/3/4 | 17 | 23 | 8 | 2018/3/29 | 13 | 27 | 7 |
| 2018/3/5 | 17 | 16 | 9 | 2018/3/30 | 18 | 24 | 10 |
| 2018/3/6 | 11 | 21 | 4 | 2018/3/31 | 12 | 20 | 6 |
| 2018/3/7 | 19 | 13 | 8 | 2018/4/1 | 9 | 8 | 2 |
| 2018/3/8 | 20 | 17 | 5 | 2018/4/2 | 7 | 5 | 1 |
| 2018/3/9 | 11 | 7 | 6 | 2018/4/3 | 9 | 2 | 2 |
| 2018/3/10 | 4 | 14 | 16 | 2018/4/4 | 4 | 3 | 2 |
| 2018/3/11 | 17 | 13 | 16 | 2018/4/5 | 4 | 10 | 4 |
| 2018/3/12 | 26 | 8 | 26 | 2018/4/6 | 4 | 10 | 11 |
| 2018/3/13 | 14 | 15 | 9 | 2018/4/7 | 3 | 20 | 5 |
| 2018/3/14 | 28 | 7 | 8 | 2018/4/8 | 11 | 9 | 6 |
| 2018/3/15 | 14 | 5 | 7 | 2018/4/9 | 15 | 11 | 9 |
| 2018/3/16 | 5 | 1 | 3 | 2018/4/10 | 15 | 25 | 9 |
| 2018/3/17 | 7 | 10 | 3 | 2018/4/11 | 12 | 8 | 12 |
| 2018/3/18 | 3 | 12 | 8 | 2018/4/12 | 18 | 19 | 14 |
| 2018/3/19 | 4 | 10 | 10 | 2018/4/13 | 28 | 62 | 39 |
| 2018/3/20 | 13 | 0 | 11 | 2018/4/14 | 16 | 43 | 19 |
| 2018/3/21 | 6 | 1 | 5 | 2018/4/15 | 15 | 7 | 1 |
| 2018/3/22 | 12 | 3 | 2 | 2018/4/16 | 6 | 0 | 2 |
| SUM | 345 | 333 | 292 | 2018/4/17 | 3 | 1 | 1 |
| % | 36% | 34% | 30% | 2018/4/18 | 22 | 10 | 1 |
|  |  |  |  | 2018/4/19 | 15 | 14 | 5 |
|  |  |  |  | 2018/4/20 | 15 | 6 | 7 |
|  |  |  |  | 2018/4/21 | 5 | 3 | 0 |
|  |  |  |  | 2018/4/22 | 2 | 5 | 2 |
|  |  |  |  | 2018/4/23 | 6 | 12 | 1 |
|  |  |  |  | 2018/4/24 | 20 | 1 | 9 |
|  |  |  |  | 2018/4/25 | 9 | 2 | 1 |
|  |  |  |  | 2018/4/26 | 13 | 2 | 1 |
|  |  |  |  | 2018/4/27 | 22 | 6 | 0 |
|  |  |  |  | 2018/4/28 | 8 | 4 | 2 |
|  |  |  |  | 2018/4/29 | 10 | 5 | 1 |
|  |  |  |  | 2018/4/30 | 30 | 2 | 7 |
|  |  |  |  | 2018/5/1 | 17 | 3 | 0 |
|  |  |  |  | 2018/5/2 | 16 | 1 | 4 |
|  |  |  |  | 2018/5/3 | 25 | 4 | 2 |
|  |  |  |  | 2018/5/4 | 15 | 5 | 12 |
|  |  |  |  | 2018/5/5 | 12 | 1 | 1 |
|  |  |  |  | 2018/5/6 | 12 | 0 | 0 |
|  |  |  |  | 2018/5/7 | 19 | 4 | 5 |
|  |  |  |  | 2018/5/8 | 18 | 4 | 2 |
|  |  |  |  | 2018/5/9 | 16 | 3 | 0 |
|  |  |  |  | 2018/5/10 | 20 | 20 | 11 |
|  |  |  |  | 2018/5/11 | 9 | 6 | 1 |
|  |  |  |  | 2018/5/12 | 10 | 4 | 5 |
|  |  |  |  | 2018/5/13 | 6 | 2 | 1 |
|  |  |  |  | 2018/5/14 | 12 | 1 | 2 |
|  |  |  |  | 2018/5/15 | 20 | 6 | 4 |
|  |  |  |  | 2018/5/16 | 10 | 8 | 2 |
|  |  |  |  | 2018/5/17 | 34 | 16 | 4 |
|  |  |  |  | SUM | 737 | 515 | 266 |
|  |  |  |  | % | 49% | 24% | 17% |

| **Department B (Before PA)** | | | | | **Department B (After PA)** | | | | |
| --- | --- | --- | --- | --- | --- | --- | --- | --- | --- |
| Date | About us | Programs | Courses and Credits | Faculty | Date | About us | Faculty | Programs | Courses and Credits |
| 2018/2/26 | 107 | 60 | 38 | 6 | 2018/3/23 | 7 | 13 | 4 | 3 |
| 2018/2/27 | 76 | 43 | 15 | 10 | 2018/3/24 | 8 | 9 | 6 | 3 |
| 2018/2/28 | 80 | 41 | 34 | 15 | 2018/3/25 | 9 | 3 | 5 | 3 |
| 2018/3/1 | 70 | 50 | 33 | 15 | 2018/3/26 | 14 | 7 | 12 | 0 |
| 2018/3/2 | 51 | 24 | 15 | 17 | 2018/3/27 | 13 | 17 | 2 | 2 |
| 2018/3/3 | 33 | 23 | 12 | 8 | 2018/3/28 | 31 | 17 | 13 | 8 |
| 2018/3/4 | 50 | 29 | 18 | 13 | 2018/3/29 | 38 | 7 | 8 | 6 |
| 2018/3/5 | 47 | 24 | 15 | 16 | 2018/3/30 | 19 | 11 | 18 | 7 |
| 2018/3/6 | 49 | 10 | 7 | 19 | 2018/3/31 | 23 | 9 | 16 | 7 |
| 2018/3/7 | 29 | 26 | 15 | 20 | 2018/4/1 | 25 | 7 | 16 | 9 |
| 2018/3/8 | 24 | 16 | 12 | 13 | 2018/4/2 | 29 | 13 | 7 | 4 |
| 2018/3/9 | 30 | 12 | 10 | 23 | 2018/4/3 | 16 | 11 | 7 | 9 |
| 2018/3/10 | 20 | 16 | 6 | 9 | 2018/4/4 | 21 | 5 | 8 | 5 |
| 2018/3/11 | 20 | 8 | 12 | 11 | 2018/4/5 | 13 | 3 | 5 | 3 |
| 2018/3/12 | 20 | 15 | 11 | 10 | 2018/4/6 | 26 | 6 | 10 | 16 |
| 2018/3/13 | 33 | 13 | 11 | 15 | 2018/4/7 | 23 | 4 | 4 | 5 |
| 2018/3/14 | 58 | 32 | 19 | 25 | 2018/4/8 | 27 | 5 | 11 | 9 |
| 2018/3/15 | 25 | 23 | 17 | 13 | 2018/4/9 | 21 | 11 | 11 | 6 |
| 2018/3/16 | 28 | 19 | 8 | 8 | 2018/4/10 | 19 | 9 | 13 | 7 |
| 2018/3/17 | 29 | 6 | 6 | 7 | 2018/4/11 | 19 | 10 | 8 | 7 |
| 2018/3/18 | 16 | 9 | 16 | 4 | 2018/4/12 | 5 | 6 | 7 | 0 |
| 2018/3/19 | 14 | 4 | 8 | 16 | 2018/4/13 | 14 | 6 | 3 | 1 |
| 2018/3/20 | 8 | 5 | 2 | 16 | 2018/4/14 | 10 | 18 | 1 | 0 |
| 2018/3/21 | 11 | 6 | 6 | 6 | 2018/4/15 | 12 | 5 | 5 | 2 |
| 2018/3/22 | 16 | 27 | 5 | 14 | 2018/4/16 | 8 | 13 | 4 | 2 |
| SUM | 944 | 541 | 351 | 329 | 2018/4/17 | 11 | 10 | 9 | 1 |
| % | 44% | 25% | 16% | 15% | 2018/4/18 | 10 | 12 | 4 | 3 |
|  |  |  |  |  | 2018/4/19 | 19 | 19 | 5 | 4 |
|  |  |  |  |  | 2018/4/20 | 32 | 26 | 13 | 20 |
|  |  |  |  |  | 2018/4/21 | 51 | 19 | 23 | 12 |
|  |  |  |  |  | 2018/4/22 | 12 | 6 | 2 | 1 |
|  |  |  |  |  | 2018/4/23 | 18 | 10 | 2 | 3 |
|  |  |  |  |  | 2018/4/24 | 10 | 2 | 7 | 3 |
|  |  |  |  |  | 2018/4/25 | 5 | 10 | 3 | 2 |
|  |  |  |  |  | 2018/4/26 | 9 | 6 | 3 | 2 |
|  |  |  |  |  | 2018/4/27 | 11 | 7 | 11 | 1 |
|  |  |  |  |  | 2018/4/28 | 7 | 2 | 5 | 2 |
|  |  |  |  |  | 2018/4/29 | 13 | 1 | 4 | 7 |
|  |  |  |  |  | 2018/4/30 | 10 | 8 | 2 | 0 |
|  |  |  |  |  | 2018/5/1 | 23 | 17 | 6 | 5 |
|  |  |  |  |  | 2018/5/2 | 4 | 16 | 7 | 4 |
|  |  |  |  |  | 2018/5/3 | 3 | 7 | 6 | 5 |
|  |  |  |  |  | 2018/5/4 | 11 | 5 | 9 | 4 |
|  |  |  |  |  | 2018/5/5 | 8 | 4 | 3 | 2 |
|  |  |  |  |  | 2018/5/6 | 5 | 1 | 2 | 1 |
|  |  |  |  |  | 2018/5/7 | 2 | 4 | 2 | 0 |
|  |  |  |  |  | 2018/5/8 | 11 | 16 | 11 | 2 |
|  |  |  |  |  | 2018/5/9 | 4 | 4 | 4 | 3 |
|  |  |  |  |  | 2018/5/10 | 5 | 6 | 7 | 4 |
|  |  |  |  |  | 2018/5/11 | 10 | 6 | 16 | 3 |
|  |  |  |  |  | 2018/5/12 | 7 | 2 | 1 | 1 |
|  |  |  |  |  | 2018/5/13 | 7 | 2 | 3 | 0 |
|  |  |  |  |  | 2018/5/14 | 10 | 15 | 3 | 2 |
|  |  |  |  |  | 2018/5/15 | 15 | 7 | 2 | 2 |
|  |  |  |  |  | 2018/5/16 | 3 | 13 | 9 | 2 |
|  |  |  |  |  | 2018/5/17 | 16 | 11 | 8 | 5 |
|  |  |  |  |  | SUM | 812 | 499 | 396 | 230 |
|  |  |  |  |  | % | 42% | 26% | 20% | 12% |
